# Common carotid artery wall thickness and external diameter as predictors of prevalent and incident cardiac events in a large population study

**DOI:** 10.1186/1476-7120-5-11

**Published:** 2007-03-09

**Authors:** Marsha L Eigenbrodt, Rishi Sukhija, Kathryn M Rose, Richard E Tracy, David J Couper, Gregory W Evans, Zoran Bursac, Jawahar L Mehta

**Affiliations:** 1 Department of Epidemiology, Fay W. Boozman College of Public Health, University of Arkansas for Medical Sciences, Little Rock, AR, USA; 2 Department of Internal Medicine, Division of Cardiovascular Medicine, University of Arkansas for Medical Sciences, Little Rock, AR, USA; 3 Department of Epidemiology, School of Public Health, University of North Carolina at Chapel Hill, NC, USA; 4 Department of Pathology, Louisiana State University Health Science Center, New Orleans, LA, USA; 5 Department of Biostatistics, University of North Carolina at Chapel Hill, NC, USA; 6 Department of Biostatistical Sciences, Division of Public Health Sciences and Department of Neurology, Wake Forest University School of Medicine, Winston-Salem, NC, USA; 7 Department of Biostatistics, Fay W. Boozman College of Public Health, University of Arkansas for Medical Sciences, Little Rock, AR, USA; 8 Department of Internal Medicine, Division of Cardiovascular Medicine, and Departments of Physiology and Biophysics, University of Arkansas for Medical Sciences, Little Rock, AR, USA

## Abstract

**Background:**

Arterial diameters enlarge in response to wall thickening, plaques, and many atherosclerotic risk factors. We hypothesized that right common carotid artery (RCCA) diameter would be independently associated with cardiac disease and improve risk discrimination.

**Methods:**

In a middle-aged, biracial population (baseline n = 11225), we examined associations between 1 standard deviation increments of baseline RCCA diameter with prevalent myocardial infarction (MI) and incident cardiac events (MI or cardiac death) using logistic regression and Cox proportional hazards models, respectively. Areas under the receiver operator characteristic curve (AUC) were used to estimate model discrimination.

**Results:**

MI was present in 451 (4%) participants at baseline (1987–89), and incident cardiac events occurred among 646 (6%) others through 1999. Adjusting for IMT, RCCA diameter was associated with prevalent MI (female OR = 2.0, 95%CI = 1.61–2.49; male OR = 1.16, 95% CI = 1.04–1.30) and incident cardiac events (female HR = 1.75, 95% CI = 1.51–2.02; male HR = 1.27, 95% CI = 1.15–1.40). Associations were attenuated but persisted after adjustment for risk factors (not including IMT) (prevalent MI: female OR = 1.73, 95% CI = 1.40–2.14; male OR = 1.14, 95% CI = 1.02–1.28, and incident cardiac events: female HR = 1.26, 95% CI = 1.08–1.48; male HR = 1.19, 95% CI = 1.08–1.32). After additional adjustment for IMT, diameter was associated with incident cardiac events in women (HR = 1.18, 95% CI = 1.00–1.40) and men (HR = 1.17, 95% CI = 1.06–1.29), and with prevalent MI only in women (OR = 1.73; 95% CI = 1.37–2.17). In women, when adjustment was limited, diameter models had larger AUC than other models.

**Conclusion:**

RCCA diameter is an important correlate of cardiac events, independent of IMT, but adds little to overall risk discrimination after risk factor adjustment.

## Background

Early identification of persons at high risk of cardiac events would improve preventive and clinical care [1]. Therefore, many novel risk factors, markers [2,3] and innovative techniques including 3-dimensional vascular measurements have been developed [4-7] that investigate intervening steps in the atherosclerosis progression. B-mode ultrasound measurement of common carotid artery (CCA) intima media thickness (IMT) is a widely available, relatively inexpensive, highly standardized method for evaluating presence and severity of atherosclerosis [8]. CCA IMT has been related to multiple atherosclerosis risk factors [9,10] and to prevalent and incident coronary heart disease (CHD) [11-16]. CCA diameter, like IMT, is easily assessed by B-mode ultrasound and is related to many atherosclerosis risk factors [9,17-20]. Larger external CCA diameter has been positively related to coronary artery disease status [21], and with acute coronary syndrome [22]. Because arterial diameter enlargement occurs early in atherosclerosis [23] and is exaggerated in the presence of vulnerable plaques [24-27], CCA diameter might improve our understanding of atherosclerosis progression and improve prediction of cardiovascular events [19] or vascular injury [28]. While 3-dimensional artery measurements have proven very useful in evaluating atherosclerosis progression[29], 2-dimensional studies have not consistently shown improvement compared to IMT [28,30-32]. We proposed to determine whether B-mode ultrasound right CCA (RCCA) diameter, or wall area calculated from 2-dimensional ultrasound measures, provides information relevant to prevalent and incident cardiac disease in addition to that provided by IMT.

## Materials and methods

### Atherosclerosis Risk in Communities Limited Access Data and Risk Factor Measurements

We examined the ARICLAD database, a subset of the Atherosclerosis Risk in Communities (ARIC) Study data consisting of participants who consented to data sharing (99.6% of participants). Details regarding the purpose and design of the ARIC Study have been published [33]. ARIC is a prospective study of 15,792 men and women, ages 45–64 at baseline, who were randomly selected from Forsyth County, North Carolina; Jackson, Mississippi; suburbs of Minneapolis, Minnesota; and Washington County, Maryland. Participants in Jackson were exclusively black; participants in Minnesota and Washington County were predominantly non-black (white); and about 14% of Forsyth County participants were black. The baseline clinic examination (exam 1) took place during 1987–1989 and three follow-up exams were conducted at approximately 3-year intervals. Surveillance is ongoing in the ARIC study [34]. For the current study we used the Limited Access Data (LAD), for which follow-up for incident cardiac events is available through December 31, 1999.

The methods for the ARIC laboratory (white blood cell count, glucose, LDL cholesterol, HDL cholesterol, and fibrinogen) and other measurement procedures (blood pressure, height and weight) have been summarized previously [35,36]. Prevalent diabetes mellitus was defined as a fasting glucose level = 7.0 mmol/L (126 mg/dl), a non-fasting level = 11.1 mmol/L (200 mg/dl), self-reported physician diagnosis of diabetes, or use of medication for diabetes within 2 weeks. The means of the second and third values for sitting blood pressures (taken with a standard clinical sphygmomanometer after a 5 minute rest) were used. Hypertension was defined as blood pressure ≥ 140/90 mm Hg or anti-hypertensive medication use within 2 weeks. Current smoking and current alcohol intake were based on self-report. For amount of alcohol consumed, the usual number of drinks per week were converted to grams/week [37]. Body mass index (BMI) was calculated as weight in kilograms/height in meters^2^.

### Prevalent and Incident Cardiac Events

Baseline prevalent myocardial infarction (MI) was based on self-reported physician diagnosis of a heart attack or changes on baseline study electrocardiogram (ECG). Incident cardiac events were ARIC adjudicated, definite or probable MI from hospital surveillance, silent MI on a follow-up study ECG, or definite fatal coronary heart disease (CHD) from surveillance. Through annual telephone interviews and surveillance of all area hospitals [34] possible cardiac events and deaths were identified for review by trained abstractors. Chest pain, ECG, and cardiac enzyme levels were used to categorize hospital events as definite or probable MI. In-hospital deaths were categorized as definite fatal CHD based on chest pain symptoms, underlying cause of death from the death certificate, and relevant hospital and ARIC clinic information. Out of hospital deaths were based on death certificates, and information from the next of kin, the patient's physician, the coroner, and autopsy or coroner reports, if available. We use the term CHD to define a broader category of cardiac events that also included cardiac procedures (coronary bypass and angioplasty). The AUCs were determined for incident events occurring within 10 years of follow-up.

### B-mode Ultrasound Measurements

The ARIC ultrasound procedures were performed by trained technicians using a Biosound 2000II Ultrasound Imaging System[38]. As previously described [39,40], at the baseline exam, each field center followed a standard procedure where scans of three longitudinal views of the carotids were recorded with the patient in a supine position. Baseline scans were read later at a central reading center [39]. The "optimal" view was the longitudinal view with a visible flow divider and a visible carotid bulb origin and was usually 10° to 20° above horizontal. Two additional views were taken at 55° above horizontal (anterior) and 10° below horizontal (posterior). This study used means of all available far wall IMT and external diameter measurements from the distal 1 cm segment of the RCCA where up to 11 measurements were possible. External diameters did not include adventitia but were the distances between the near and far wall media-adventitial interfaces. Plaques were not purposefully excluded from CCA measurements [40]. In this study RCCA measurements were used because of more complete information [41] and because of similarity of risk factor associations for right and left CCA diameters [9]. RCCA round and elliptical wall areas calculations used in this study have been previously described [42].

### Study Population

From the 15,732 participants in the ARICLAD, 10000 were randomly selected as a developmental data set with the remainder (5732) reserved as a test data set. Group assignment was made prior to exclusions because the samples were to be used in a series of studies requiring different exclusions. Participants were excluded if the following information was missing: baseline prevalent MI or CHD status (n = 347), ultrasound vascular measures needed for wall area calculations (n = 3620), or data on covariates (n = 540). The total effective baseline sample was 11225 with a developmental data set of 7125 (3215 men and 3910 women) and a test data set of 4100 persons (1846 men and 2254 women). Excluding 451 participants (347 men and 104 women) with a baseline MI, left 10774 (4714 men and 6060 women) for incident event analyses.

### Statistical Analysis

All statistical analyses were performed with SAS version 8.2 or 9.1. The cross-sectional and prospective associations for RCCA ultrasound measures with prevalent and incident cardiac events were evaluated in the full sample using logistic regression and Cox proportional hazards models, respectively. One standard deviation (SD) of each RCCA measure in the developmental data set served as the unit of measure (IMT = 0.18 mm, diameter = 0.96 mm, and wall area = 4.9 mm^2^). Because gender was an effect modifier (interaction term p < 0.05) of the diameter-cardiac event relationship, gender-specific models were developed. Age was an effect modifier for vascular associations with incident cardiac events among men (interaction term p < 0.05); thus, age-specific values were determined for men.

Unadjusted models contained one of the following: IMT, diameter, calculated wall area, or IMT + diameter. "Demographic" models added demographic variables of age and ethnicity as well as height. Risk-adjusted models included cardiovascular risk factors chosen from the developmental data set by stepwise logistic regression analyses after forcing age and race into the gender-specific models. Potential covariates included height (nearest cm), body mass index (BMI) (weight in kg/height in m^2^), diabetes mellitus, hypertension, LDL- and HDL-cholesterol (mmol/L), systolic and diastolic blood pressures (mm Hg), current smoking status, current drinking status and ethanol consumption (grams/week), white blood cell count (1000s / mm^3^), use of cholesterol lowering medication, glucose (mg/dl), and fibrinogen (mg/dl). Covariates were identified separately for prevalent and incident cardiac events. All covariates that were significant (p ≤ 0.05) in any of the single vascular models (IMT, diameter, or wall areas) were included so that models being compared for specific outcomes differed only in the vascular measure(s) that were included. The AUC for both developmental and test data sets were based on the risks estimated for each individual using coefficients identified from regression models for prevalent MI and incident cardiac events [36]. The final model was stratified by baseline plaques/shadowing status with CHD as the outcome.

Median values of IMT and diameter were used as the cut points to categorize individuals as having large and not large RCCA IMT and diameter (men: IMT 0.66 mm and diameter 8.08 mm; women: IMT 0.62 mm and diameter 7.31 mm). Gender-specific event free survival functions were plotted for the four IMT-diameter groups: large or "normal" (not large) IMT and large or "normal" diameter without further adjustment.

## Results

The characteristics of the participants randomized to the developmental and test data sets were similar. Only 1 characteristic (diabetes mellitus), out of 23 (data not shown), differed significantly (p = 0.05) between the developmental and test data sets (developmental data set diabetes prevalence = 11.0%; test data set prevalence = 9.8%, p = 0.04). Thus, random assignment of participants into developmental and test data sets appeared successful.

### Baseline Characteristics

MI was identified at baseline in 289 (4.1%) of the 7125 persons in the developmental data set, and in 162 (4.0%) of the 4100 individuals in the test data set. Incident cardiac events occurred in 3.8% of women and 8.8% of men through 1999. Characteristics of the study population at baseline, overall and by baseline MI status, are provided in Table 1. Participants with MI at baseline were older and had a significantly higher prevalence of several cardiovascular risk factors (male gender, diabetes, hypertension, current smoking and non-drinking) than those without an MI. Detrimental cross-sectional associations were also found for several continuous risk factors with BMI, LDL, fasting glucose, white blood count, and fibrinogen being higher, and HDL being lower in persons with, compared to those without MI. Persons with prevalent MI had significantly thicker RCCA IMT and larger RCCA diameter and RCCA wall areas than persons without MI.

**Table 1:** Baseline characteristics for the developmental data set and for subsets with and without prevalent myocardial infarction at baseline, ARICLAD, 1987–89

		Baseline Myocardial Infarction	
	Developmental Sample	Absent	Present

	N = 7125	N = 6836	N = 289

Age in years	54.2 ± 5.7	54.1 ± 5.7	56.9 ± 5.1***
Gender (% male)	45.1	43.8	76.1***
Race (% nonwhite)	25.9	25.9	23.9
Diabetes (%)	9.8	9.2	23.2***
Hypertension (%)	33.0	32.2	52.6***
Height in cm	168.5 ±9.3	168.4 ± 9.3	172.1 ± 8.0***
BMI (kg/m^2^)	27.2 ± 5.0	27.1 ± 5.0	27.9 ± 5.2**
LDL-C (mmol/L)	3.55 ±1.02	3.54 ± 1.02	3.78 ± 1.03***
HDL-C (mmol/L)	1.36 ± 0.45	1.37 ± 0.45	1.11 ± 0.38***
Cholesterol Med. (% use)	2.6	2.5	5.2**
Fasting glucose (mg%)^†^	106.7 ± 36.2	106.2 ± 35.6	117.3 ± 46.5***
White blood count (1000s/ml)	6.10 ± 1.94	6.08 ± 1.93	6.74 ± 1.99***
Fibrinogen (mg/dl)^‡^	302.4 ± 65.2	301.4 ± 64.8	327.2 ± 70.8***
Current drinker (%)	57.2	57.5	49.1**
Current smoker (%)	27.2	27.0	32.5*
Systolic BP (mm Hg)	121.0 ± 18.7	120.9 ± 18.6	121.7 ± 21.1
Diastolic BP (mm Hg)	73.4 ± 11.3	73.5 ± 11.2	72.1 ± 12.2
RCCA IMT in mm	0.66 ± 0.18	0.66 ± 0.17	0.74 ± 0.25***
RCCA diameter in mm	7.78 ± 0.95	7.76 ± 0.94	8.32 ± 1.08***
RCCA wall area in mm^2^	14.97 ± 4.94	14.85 ± 4.81	17.83 ± 6.73***

Values are expressed as percent or as means ± standard deviation

ARICLAD: Atherosclerosis Risk in Communities Limited Access Data

BMI: body mass index= weight in kg/height in meters^2^

BP: blood pressure

HDL- and LDL-cholesterol: high and low density lipoprotein cholesterol

IMT: intima medial thickness

RCCA: right common carotid artery

^† ^Conversion factor = 0.0555 to convert to mmol/L

^‡^Conversion factor = 0.0294 to convert to μmol/L

*p-values = 0.05 **p-value = 0.005 ***p-value = 0.0005 for chi-square and Wilcoxon two-sample test for difference in the characteristic by baseline myocardial infarction status

### Association of Vascular Measures with Prevalent and Incident Cardiac Events

For both men and women, when evaluated in separate models, RCCA IMT, diameter, and wall area were positively associated with baseline prevalent MI (Table 2) and with incident cardiac events (Tables 3, and 4) and generally remained statistically significant after risk factor adjustment. Among women, for both prevalent MI (Table 2) and incident cardiac events (Table 3), associations were stronger for diameter than IMT, and wall area associations were of intermediate strength. Risk factor adjustment attenuated diameter's effect to a greater degree for incident than prevalent events (74.3% vs. 33.6% risk reduction respectively) among women. Among men, there was little disparity in the overall strengths of the vascular measure associations for either prevalent (Table 2) or incident events (Table 4). IMT, diameter, and wall area associations with incident cardiac events were stronger among men who were 54 years of age or younger compared to older men (Table 4).

**Table 2:** Baseline cross-sectional associations between B-mode ultrasound RCCA measures and prevalent MI and the discrimination of the respective models in the developmental and test data sets of the ARICLAD, 1987–89

		Vascular Measures' Association with Prevalent MI		Discrimination of Prevalent MI			
				Men, Data Sets		Women, Data Sets	

Model	RCCA Measure	Men N = 5061 OR (95% CI)*	Women N = 6164 OR (95% CI)*	Development N = 3215 AUC	Test N = 1846 AUC	Development N = 3910 AUC	Test N = 2254 AUC

1	IMT	1.32 (1.22–1.43)	1.36 (1.18–1.55)	0.586	0.616	0.631	0.632
2	IMT	1.23 (1.13–1.34)	1.21 (1.02–1.42)	0.640	0.676	0.681	0.682
3	IMT	1.17 (1.07–1.28)	1.17 (0.99–1.38)	0.753	0.749	0.741	0.690
1	Diameter	1.29 (1.16–1.43)	2.10 (1.73–2.55)	0.551	0.614	0.689	0.687
2	Diameter	1.21 (1.08–1.35)	1.81 (1.47–2.24)	0634	0.680	0.717	0.719
3	Diameter	1.14 (1.02–1.28)	1.73 (1.40–2.14)	0.750	0.747	0.766	0.718
1	Wall area	1.36 (1.25–1.48)	1.60 (1.38–1.86)	0.585	0.638	0.678	0.675
2	Wall area	1.27 (1.16–1.39)	1.40 (1.19–1.66)	0.639	0.684	0.700	0.697
3	Wall area	1.20 (1.09–1.32)	1.35 (1.14–1.60)	0.753	0.751	0.753	0.700

Model	Two Measures	Men OR (95% CI)	Women OR (95% CI)	Men		Women	
				Developmental	Test	Developmental	Test

1	IMT + Diameter	1.27 (1.16–1.38) 1.16 (1.04–1.30)	1.09 (0.91–1.3) 2.00 (1.61–2.49)	0.586	0.644	0.696	0.687
2	IMT + Diameter	1.19 (1.09–1.31) 1.13(1.01–1.27)	1.02 (0.84–1.24) 1.80 (1.43–2.25)	0.640	0.689	0.719	0.717
3	IMT + Diameter	1.15 (1.05–1.26) 1.09 (0.97–1.23)	1.01 (0.83–1.22) 1.73 (1.37–2.17)	0.753	0.750	0.767	0.716

OR: odds ratio for 1 standard deviation increment of the vascular measure

CI: confidence interval

Model 1: vascular measure(s) only; Model 2: add age, race, and height; Model 3 for men: age, race, diabetes mellitus, systolic blood pressure, fibrinogen, HDL and LDL cholesterol, and hypertension; Model 3 for women: age, race, current drinking status, HDL cholesterol, diastolic blood pressure and white blood count.

**Table 3:** Among women, the risk associated with RCCA B-mode ultrasound vascular measures and the model discrimination in the developmental and test data sets of the ARICLAD, 1987–99

		Incident Cardiac Events		
		Vascular Measure Risk	Model Discrimination	
		Overall	Developmental	Test
Model	RCCA Measure	N = 6060	N = 3841	N = 2219
		
		HR (95% CI)	AUC	AUC
1	IMT	1.49 (1.38–1.61)	0.621	0.608
2	IMT	1.40 (1.28–1.53)	0.682	0.675
3	IMT	1.19 (1.08–1.31)	0.830	0.805
1	diameter	2.01 (1.76–2.29)	0.696	0.679
2	diameter	1.83 (1.59–2.11)	0.724	0.712
3	diameter	1.26 (1.08–1.48)	0.831	0.812
1	Wall area	1.65 (1.52–1.78)	0.680	0.661
2	Wall area	1.56 (1.42–1.71)	0.702	0.699
3	Wall area	1.23 (1.10–1.38)	0.831	0.808

Model	Two Measures	HR (95% CI)	Developmental	Test
			AUC	AUC

1	IMT + diameter	1.26 (1.15–1.39) 1.75 (1.51–2.02)	0.705	0.674
2	IMT + diameter	1.23 (1.10–1.37) 1.65 (1.42–1.93)	0.724	0.705
3	IMT + diameter	1.14 (1.03–1.27) 1.18 (1.00–1.40)	0.831	0.805

HR = hazards ratio for 1 standard deviation increment of the vascular measure in Cox proportional hazards models. CI: confidence interval

Model 1: vascular measure(s) only; Model 2 covariates: age, race, and height;

Model 3 covariates: age, race, systolic blood pressure, diabetes mellitus, HDL and LDL cholesterol, blood glucose, fibrinogen, current drinking, and current smoking.

**Table 4:** Among men, the overall and age-specific risks associated with RCCA B-mode ultrasound vascular measures and the discrimination for the overall models in the developmental and test data sets of the ARICLAD, 1987–99

		Risk for Incident Cardiac Events			Model Discrimination for All Ages	
Model	RCCA Measure	All N = 4714	Age ≤ 54 N = 2415	Age > 54 N = 2299	Development N = 2995	Test N = 1719
		
		HR (95% CI)	HR (95% CI)	HR (95% CI)	AUC	AUC
1	IMT	1.26 (1.17–1.34)	1.36 (1.23–1.50)	1.14 (1.04–1.25)	0.597	0.606
2	IMT	1.20 (1.11–1.29)	1.35 (1.22–1.50)	1.11 (1.01–1.23)	0.626	0.633
3	IMT	1.10 (1.02–1.18)	1.22 (1.10–1.36)	1.04 (0.94–1.14)	0.718	0.710
1	diameter	1.36 (1.24–1.49)	1.40 (1.21–1.62)	1.26 (1.13–1.42)	0.599	0.608
2	diameter	1.30 (1.18–1.42)	1.40 (1.20–1.62)	1.24 (1.10–1.40)	0.629	0.633
3	diameter	1.19 (1.08–1.32)	1.28 (1.09–1.49)	1.16 (1.02–1.31)	0.719	0.713
1	Wall area	1.33 (1.24–1.42)	1.45 (1.30–1.61)	1.20 (1.09–1.29)	0.614	0.636
2	Wall area	1.26 (1.17–1.37)	1.41 (1.29–1.61)	1.17 (1.06–1.29)	0.636	0.653
3	Wall area	1.15 (1.06–1.24)	1.29 (1.15–1.45)	1.08 (0.98–1.20)	0.721	0.711

Model	Double vascular	All	Age ≤ 54	Age >54		

1	IMT + diameter	1.18 (1.09–1.27) 1.27 (1.15–1.40)	1.28 (1.14–1.44) 1.26 (1.07–1.48)	1.08 (0.97–1.20) 1.23 (1.09–1.39)	0.616	0.636
2	IMT + diameter	1.13 (1.05–1.23) 1.24 (1.12–1.37)	1.27 (1.14–1.43) 1.26 (1.07–1.49)	1.05 (0.95–1.18) 1.22 (1.08–1.38)	0.637	0.652
3	IMT + diameter	1.06 (0.98–1.15) 1.17 (1.06–1.29)	1.17 (1.04–1.32) 1.20 (1.02–1.42)	1.01 (0.91–1.12) 1.16 (1.02–1.31)	0.721	0.713

HR = hazards ratio for 1 standard deviation increment of the vascular measure

Model 1: vascular measure(s) only; Model 2 covariates: age, race, and height; Model 3 covariates: age, race, BMI, HDL and LDL cholesterol, diabetes mellitus, fibrinogen, hypertension, and current smoking.

### Cardiac Events Including Procedures

The association with prevalent and incident CHD (including coronary procedures) among women was stronger for RCCA diameter than IMT, regardless of plaque/shadowing status (Table 5). RCCA wall area produced an effect intermediate between IMT and diameter among women. Among men, the IMT associations were similar to the IMT associations found among women. However, the diameter and wall area associations for men were much weaker for both prevalent and incident CHD than the associations found for women. For women the diameter associations were generally stronger in the absence of plaques/shadowing. Risk factor adjustment consistently reduced diameter associations for incident CHD in both genders regardless of baseline plaques/shadowing status, but adjustment produced less consistent attenuation for prevalent CHD.

**Table 5:** Relationships between RCCA ultrasound measures and prevalent and incident CHD among men and women with and without carotid plaques/shadowing^†^at baseline, ARICLAD

		Prevalent Coronary Heart Disease, 1987–1989				Incident Coronary Heart Disease to 12/1999			
		Men OR, 95% CI^§ ^ Plaques/Shadowing:		Women OR, 95% CI^§ ^ Plaques/Shadowing:		Men HR, 95% CI^§ ^ Plaques/Shadowing:		Women HR, 95% CI^§ ^ Plaques/Shadowing:	

Model	RCCA Measure	No (n = 2277)	Yes (n = 1867)	No (n = 3550)	Yes (n = 1657)	No (n = 2150)	Yes (n = 1677)	No (n = 3503)	Yes(n = 1603)

2^‡^	IMT	1.16	1.23	1.27	1.21	1.21	1.15	1.18	1.27
		0.94–1.44	1.10–1.37	0.88–1.84	1.01–1.45	1.02–1.42	1.06–1.25	0.92–1.52	1.13–1.42
3^‡^	IMT	1.13	1.19	1.29	1.17	1.10	1.07	1.00	1.09
		0.90–1.42	1.07–1.33	0.90–1.86	0.97–1.41	0.93–1.30	0.99–1.16	0.78–1.28	0.96–1.23
2^‡^	Diameter	1.27	1.10	1.91	1.50	1.17	1.22	1.83	1.60
		1.05–1.54	0.95–1.27	1.35–2.71	1.13–1.98	1.00–1.36	1.10–1.36	1.45–2.30	1.35–1.91
3^‡^	Diameter	1.30	1.01	1.89	1.44	1.10	1.14	1.25	1.22
		1.06–1.59	0.87–1.19	1.34–2.68	1.08–1.93	0.93–1.29	1.02–1.28	0.97–1.60	1.00–1.49
2^‡^	Wall Area	1.23	1.24	1.59	1.31	1.24	1.20	1.62	1.37
		1.00–1.52	1.11–1.39	1.11–2.29	1.07–1.61	1.05–1.45	1.10–1.32	1.29–2.03	1.22–1.54
3^‡^	Wall Area	1.23	1.18	1.63	1.26	1.13	1.11	0.88	1.12
		0.99–1.54	1.05–1.34	1.13–2.35	1.02–1.56	0.96–1.34	1.02–1.22	0.61–1.26	0.97–1.28

Model	Two Measures								

2^‡^	IMT	1.08	1.23	1.01	1.12	1.16	1.11	0.94	1.14
		0.86–1.36	1.10–1.37	0.67–1.50	0.91–1.37	0.98–1.38	1.01–1.22	0.72–1.24	1.00–1.30
	+ Diameter	1.24	1.01	1.97	1.44	1.11	1.18	1.87	1.50
		1.01–1.52	0.86–1.18	1.33–2.92	1.05–1.97	0.95–1.31	1.05–1.32	1.45–2.40	1.24–1.81
3^‡^	IMT	1.05	1.20	1.06	1.09	1.08	1.05	0.92	1.04
		0.82–1.33	1.07–1.34	0.71–1.57	0.89–1.34	0.91–1.28	0.96–1.15	0.70–1.20	0.91–1.20
	+ Diameter	1.29	0.96	1.86	1.38	1.08	1.13	1.29	1.19
		1.04–1.59	0.81–1.12	1.28–2.70	1.02–1.88	0.91–1.28	1.00–1.27	0.99–1.68	0.96–1.48

^§^Odds ratios (OR) or hazards ratio (HR) associated with an increment of 1 standard deviation of the vascular measure in the logistic and Cox proportional hazards models respectively

^†^Plaques or shadowing at any right or left carotid site (internal, CCA, or bifurcation)

^‡^Models 2 and 3 are demographic and risk factor adjusted models respectively. The covariates for prevalent disease are found in Table 2 and those for incident events are listed in Table 3 for women and Table 4 for men.

CHD, coronary heart disease that includes coronary artery procedures.

### Model Discrimination for Prevalent and Incident Cardiac Events

The receiver operator characteristic curve plots the sensitivity vs. 1 – specificity and the AUC is the associated measure of the model's discrimination of the outcome for individuals [36]. For men, unadjusted model discrimination varied between the developmental and test data sets (Tables 2 and 4). Neither IMT nor diameter provided consistently better discrimination. Wall area or IMT+diameter provided more consistent discrimination for incident and prevalent events across subsets of men than either IMT or diameter alone.

The AUCs were consistently larger for women than men in both prevalent and incident analyses (Tables 2 and 3). Among women when adjustment was limited, models with diameter had consistently larger AUC than models with IMT in both developmental and test data sets for prevalent (Table 2 right: AUC differences ≈ 0.06 for unadjusted models, ≈ 0.04 for demographic models) and incident models (Table 3 right: AUC differences ≈ 0.07 for unadjusted and ≈ 0.04 for demographic models). After risk factor adjustment, little difference remained for prevalent MI (Table 2: AUC differences ≈ 0.03) and incident cardiac events (Table 3: AUC differences ≈ 0.001 and 0.007 in developmental and test data sets respectively). Wall area models were generally intermediate in discrimination between IMT and diameter models.

**Figure 1:**
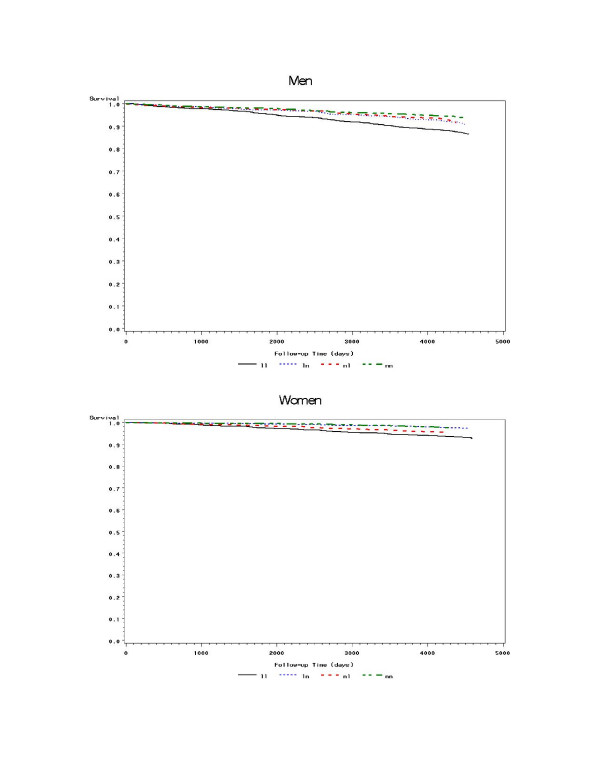
**a **and **b**: Gender-specific survival functions for four IMT-diameter groups, unadjusted for risk factors. Black solid line (ll) indicates large IMT and large diameter group; blue dashed line (ln) indicates large IMT and normal diameter group; red dotted line (nl) indicates normal IMT and large diameter group; green dot-dash line (nn) indicates normal IMT and normal diameter group.

For both men and women (Figures 1a and 1b), those who had both large diameter and large IMT had the poorest event-free survival of all four IMT-diameter groups. For men, the remaining three categories diverged only modestly and only late in follow up. Women with large diameters but normal IMT had the second poorest survival of the four groups.

## Discussion

The current study provides evidence suggesting the importance of measuring arterial diameter, as well as IMT, in studies of atherosclerosis. The largest reduction in event free survival among both men and women occurred among persons having diameter enlargement as well as thickened IMT. Changes in diameter effect size after adjustment for IMT and risk factors suggest that risk factors contribute to cardiac events through diameter and IMT changes.

The present study confirmed an association between RCCA diameter and prevalent and incident cardiac events for both men and women with evidence of independence from many traditional risk factors when IMT and diameter were evaluated separately. Among women, even with RCCA IMT included as a risk factor, diameter was independently associated with incident cardiac events; and models for women with diameter alone or diameter + IMT provided generally better discrimination than models with IMT alone. In evaluations of prevalent and incident cardiac events for men, model discrimination was generally more consistent across test and developmental subsets when RCCA wall area or both IMT and diameter were included in the models. Both men and women with enlarged diameter and IMT had the poorest event-free survival. Since plaque prevalence has been associated with cardiovascular events even after control for IMT [14], our hypothesis was that diameter would improve prediction of cardiac events by providing an indication of the presence and severity of plaques. However, diameter may indicate not only plaque presence [43] or vulnerability [26,27], but also responses to hemodynamic factors [44,45], to aging [41,46], to the metabolic syndrome [20], or to other factors. The stronger association with cardiac events in the absence of plaques in our study supports the view that diameter indicates more than just plaque presence/severity.

It should not be surprising that IMT and diameter may have independent effects on both prevalent and incident cardiac events given that IMT and diameter have somewhat different risk factor relationships [9,13,17,28,47]. Thus, conditions related to diameter such as the metabolic syndrome [20] or novel risk factors [48,49] may lead to diameter enlargement early in the atherosclerotic process. Later, outward vascular remodeling in response to atherosclerotic plaques may result in diameter enlargement. Also, arterial diameters enlarge with age [18,41]. Thus, diameter may reflect the impact of different factors depending upon the person's age and stage of atherosclerosis. Gender differences may reflect the different stage of atherosclerosis found among men and women of similar ages. The lower overall incidence of events among women contributed to the higher relative risk associated with diameter compared to men whose absolute risk was higher. It is possible that diameter changes contribute to the heterogeneity of IMT effect with age that was reported in a recent meta-analysis [50].

Artery and plaque dimensions have been previously related to cardiovascular events [19,21,28,31]. In a study of symptomatic patients, Nowak et. al. found that that left CCA intima-medial wall area was a better indicator of the extent of angiographically determined coronary artery disease than IMT [31]. In a small clinical study, patients with acute coronary syndrome had larger right and left carotid lumen diameters than persons with stable angina [22]. In our study, diameter complemented IMT for risk discrimination of cardiac events, especially with limited risk factor information. Among men, inclusion of both IMT and diameter produced discrimination of events that was more consistent across subsets than either IMT or diameter alone. Among women, diameter was a stronger risk discriminator than IMT, especially at basic levels of adjustment. While sophisticated techniques for evaluating 3-dimensional artery and plaques may provide more precise measurements [5-7,29], B-mode ultrasound is more widely available and less expensive, and its usefulness may be improved by evaluating both RCCA IMT and diameter.

Results from our study raise the question of whether differences in initial vascular size may influence arterial response to shear stress. As plaques develop and encroach on the artery lumen, arteries enlarge to maintain blood flow and levels of shear stress [51]. Larger arteries would need to enlarge less to preserve lumen area than smaller arteries [42]. Thus, the larger CCAs in healthy men than in women [41], and the larger diameters at older ages even in healthy populations, might explain the stronger diameter associations found for women compared to men and for younger versus older men. Age might also explain the minimal effect reported in the Rotterdam Study, a study of a higher risk, older population (age ≥ 55 years), where 1 SD of arterial mass (wall area) had a minimally higher HR compared to 1 SD of IMT [30], but no improvement in the AUC was seen. Thus, the effects of ectasia associated with normal aging may dilute any attempt to identify detrimental effects associated with diameter among the elderly. Thus, diameter may be a surrogate for different factors at various ages or stages of disease.

In our study, inclusion of covariates that optimized all vascular measure models, allowed an unbiased comparison of vascular models' discrimination, but prevents comparison of the AUCs with other reported predictive models that included different covariates [2,36]. A potential for misclassification by plaque status exists in the ARIC study [52], potentially impacting our analyses that were stratified by plaques/shadowing status. Finally, correlation of vascular measures and risk factors [2], and misclassification of the outcome [53] may prevent further improvement in model discrimination. While many manuscripts based on the full ARIC data use IMT measures that have been reader trend adjusted, those values are not available in the limited access data used in this study.

In conclusion, B-mode ultrasound of the carotid artery can be used to assess the presence and extent of vascular disease, even at an early stage. Our study suggests that diameter complements IMT as an indicator of atherosclerosis. Consideration of arterial diameter along with arterial wall thickness may provide insights into progression to cardiovascular disease events.

## Competing interests

The author(s) declare that they have no competing interests.

## Authors' contributions

All authors have approved the manuscript for submission. Additional contributions follow:

MLE developed the study proposal, performed prospective analyses, summarized the results, and provided extensive input into all sections of the manuscript.

RS did an early literature review and drafted the initial background section of the manuscript and provided input into other sections.

KMR provided input into the study development and provided suggestions and revised drafts of the manuscript.

RET provided insight into the process of arterial aging and comments on manuscript drafts.

DJC and GWE provided statistical input into the manuscript, provided comments on drafts of the manuscript, and GWE provided insights into the ARIC ultrasound procedures.

ZB provided initial data sets and initial cross-sectional analyses and survival curves.

JLM provided comments on several drafts of the manuscript.

## References

[B1] Grundy SM, Bazzarre T, Cleeman J, D'Agostino RB, Hill M, Houston-Miller N, Kannel WB, Krauss R, Krumholz HM, Lauer RM, Ockene IS, Pasternak RC, Pearson T, Ridker PM, Wood D (2000). Prevention Conference V: Beyond secondary prevention: identifying the high-risk patient for primary prevention: medical office assessment: Writing Group I. Circulation.

[B2] Folsom AR, Chambless LE, Ballantyne CM, Coresh J, Heiss G, Wu KK, Boerwinkle E, Mosley TH, Sorlie P, Diao G, Sharrett AR (2006). An assessment of incremental coronary risk prediction using C-reactive protein and other novel risk markers. Arch Intern Med.

[B3] van der Meer IM, Bots ML, Hofman A, del Sol AI, van der Kuip DAM, Witteman JCM (2004). Predictive value of noninvasive measures of atherosclerosis for incident myocardial infarction: the Rotterdam Study. Circulation.

[B4] Hennerici M, Baezner H, Daffertshofer M (2004). Ultrasound and arterial wall disease. Cerebrovasc Dis.

[B5] Jaffer FA, Weissleder R (2005). Molecular imaging in the clinical area. JAMA.

[B6] Crowe LA, Ariff B, Keegan J, Mohiaddin RH, Yang GZ, Hughes AD, Thom SA, Firmin DN (2005). Comparison between three-dimensional volume-selective turbo spin-echo imaging and two-dimensional ultrasound for assessing carotid artery structure and function. J Magn Reson Imaging.

[B7] Landry A, Spence JD, Fenster A (2004). Measurement of carotid plaque volume by 3-Dimensional ultrasound. Stroke.

[B8] Bots ML, Evans GW, Riley WA, Grobbee DE (2003). Carotid intima-media thickness measurements in intervention studies--design options, progression rates, and sample size considerations: a point of view. Stroke.

[B9] Crouse JR, Goldbourt U, Evans G, Pinsky J, Sharrett AR, Sorlie P, Riley W, Heiss G (1996). Risk factors and segment-specific carotid arterial enlargement in the Atherosclerosis Risk in Communities (ARIC) cohort. Stroke.

[B10] Labropoulos N, Leon LR, Brewster LP, Tiongson J, Kang SS, Mansour MA, Kalman P (2005). Are your arteries older than your age?. Eur J Vasc Endovasc Surg.

[B11] Burke GL, Evans GW, Riley WA, Sharrett AR, Howard G, Barnes RW, Rosamond W, Crow RS, Rautaharju PM, Heiss G (1995). Arterial wall thickness is associated with prevalent cardiovascular disease in middle-aged adults. The Atherosclerosis Risk in Communities (ARIC) Study. Stroke.

[B12] O'Leary DH, Polak JF, Kronmal RA, Manolio TA, Burke GL, Wolfson SK (1999). Carotid-artery intima media thickness as a risk factor for myocardial infarction and stroke in older adults. N Engl J Med.

[B13] Crouse JR, Craven TE, Hagaman AP, Bond MG (1995). Association of coronary disease with segment-specific intimal-medial thickening of the extracranial carotid artery. Circulation.

[B14] Rosvall M, Janzon L, Berglund G, Engstrom G, Hedblad B (2005). Incident coronary events and case fatality in relation to common carotid intima-media thickness. J Intern Med.

[B15] Lorenz MW, von Kegler S, Steinmetz H, Markus HS, Sitzer M (2006). Carotid intima-media thickening indicates higher vascular risk across a wide age range: Prospective data from the Carotid Atherosclerosis Progression Study (CAPS). Stroke.

[B16] Kablak-Ziembicka A, Przewlocki T, Tracz W, Pieniazek P, Musialek P, Sokolowski A (2005). Gender differences in carotid intima-media thickness in patients with suspected coronary artery disease. Am J Cardiol.

[B17] Crouse JR, Goldbourt U, Evans G, Pinsky J, Sharrett AR, Sorlie P, Riley W, Heiss G (1994). Arterial enlargement in the Atherosclerosis Risk in Communities (ARIC) cohort. In vivo quantification of carotid arterial enlargement. The ARIC Investigators. Stroke.

[B18] Schmidt-Trucksass A, Grathwohl D, Schmid A, Boragk R, Upmeier C, Keul J, Huonker M (1999). Structural, functional, and hemodynamic changes of the common carotid artery with age in male subjects. Arterioscler Thromb Vasc Biol.

[B19] Jensen-Urstad K, Jensen-Urstad M, Johansson J (1999). Carotid artery diameter correlates with risk factors for cardiovascular disease in a population of 55-year-old subjects. Stroke.

[B20] Iannuzzi A, De Michele M, Bond G, Sachetti L, Fortunato G, Salvatore F, Mattiello A, Panico S, Rubba P (2005). Carotid artery remodeling in middle-aged women with the metabolic syndrome (from the "Progetto ATENA" Study). Am J Cardiol.

[B21] Terry JG, Tang R, Espeland MA, Davis DH, Vieira JLC, Mercuri MF, Crouse JR (2003). Carotid arterial structure in patients with documented coronary artery disease and disease-free control subjects. Circulation.

[B22] Demircan S, Tekin A, Tekin G, Topcu S, Yigit F, Erol T, Katircibasi T, Sezgin AT, Baltali M, Ozin B, Muderrisoglu H (2005). Comparison of carotid intima-media thickness in patients with stable angina versus patients with acute coronary syndrome. Am J Cardiol.

[B23] Labropoulos N, Zarge J, Mansour MA, Kang SS, Baker WH (1998). Compensatory arterial enlargement is a common pathobiologic response in early atherosclerosis. Am J Surg.

[B24] Kiechl S, Willeit J (1999). The natural course of atherosclerosis. Part II: vascular remodeling. Bruneck Study Group. Arterioscler Thromb Vasc Biol.

[B25] Zureik M, Temmar M, Adamopoulos C, Bureau JM, Courbon D, Thomas F, Bean K, Touboul PJ, Ducimetiere P, Benetos A (2002). Carotid plaques, but not common carotid intima-media thickness, are independently associated with aortic stiffness. J Hypertens.

[B26] Burke AP, Kolodgie FD, Farb A, Weber D, Virmani R (2002). Morphological predictors of arterial remodeling in coronary atherosclerosis. Circulation.

[B27] Moreno PR, Purushothaman KR, Fuster V, O'Connor WN (2002). Intimomedial interface damage and adventitial inflammation is increased beneath disrupted atherosclerosis in the aorta: implications for plaque vulnerability. Circulation.

[B28] Lemne C, Jogestrand T, de Faire U (1995). Carotid intima-media thickness and plaque in borderline hypertension. Stroke.

[B29] Nissen SE, Tuzcu EM, Schoenhagen P, Brown BG, Ganz P, Vogel RA, Crowe T, Howard G, Cooper CJ, Brodie B, Grines CL, DeMaria AN (2004). Effect of intensive compared with moderate lipid-lowering therapy on progression of coronary atherosclerosis. JAMA.

[B30] Bots ML, Grobbee DE, Hofman A, Witteman JC (2005). Common carotid intima-media thickness and risk of acute myocardial infarction: the role of lumen diameter. Stroke.

[B31] Nowak J, Nilsson T, Sylven C, Jogestrand T (1998). Potential of carotid ultrasonography in the diagnosis of coronary artery disease: a comparison of exercise test and variance ECG. Stroke.

[B32] Spence JD, Eliasziw M, DiCicco M, Hackan DG, Galil R, Lohmann T (2002). Carotid plaque area: a tool for targeting and evaluating vascular preventive therapy. Stroke.

[B33] ARIC Investigators (1989). The Atherosclerosis Risk in Communities (ARIC) Study:  design and objectives. Am J Epidemiol.

[B34] White AD, Folsom AR, Chambless LE, Sharrett AR, Yang K, Conwill D, Higgins M, Williams OD, Tyroler HA (1996). Community surveillance of coronary heart disease in the Atherosclerosis Risk in Communities (ARIC) Study: Methods and initial two years' experience. J Clin Epidemiol.

[B35] Sharrett AR, Ballantyne CM, Coady SA, Heiss G, Sorlie PD, Catellier D, Patsch W (2001). Coronary heart disease prediction from lipoprotein cholesterol levels, triglycerides, lipoprotein(a), apolipoproteins A-I and B, and HDL density subfractions: The Atherosclerosis Risk in Communities (ARIC) Study.. Circulation.

[B36] Chambless LE, Folsom AR, Sharrett AR, Sorlie P, Couper D, Szklo M, Nieto FJ (2003). Coronary heart disease risk prediction in the Atherosclerosis Risk in Communities (ARIC) study. J Clin Epidemiol.

[B37] Eigenbrodt ML, Mosley TH, Hutchinson RG, Watson RL, Chambless LE, Szklo M (2001). Alcohol consumption with age: a cross-sectional and longitudinal study of the Atherosclerosis Risk in Communities (ARIC) study, 1987-1995. Am J Epidemiol.

[B38] Riley WA, Evans G, Barnes RW, Chambless LE, Bond MG, Heiss G (1991). High-resolution B-mode ultrasound reading methods in the Atherosclerosis Risk in Communities (ARIC) Cohort. J Neuroimaging.

[B39] National Heart, Lung, and Blood Institute. Atherosclerosis Risk in Communities (ARIC) Study. Operations manual no. 6A:ultrasound assessment--part A, ultrasound scanning, Version 1.0..

[B40] National Heart, Lung, and Blood Institute. Atherosclerosis Risk in Communities (ARIC) Study. Operations manual no. 6B:ultrasound assessment--part B, ultrasound reading, Version 1.0.

[B41] Eigenbrodt ML, Bursac Z, Rose KM, Couper DJ, Tracy RE, Evans GW, Brancati FL, Mehta JL (2006). Common carotid arterial interadventitial distances (diameter) as an indicator of the damaging effects of age and atherosclerosis, a cross-sectional study of the Atherosclerosis Risk in Community Cohort Limited Access Data (ARICLAD), 1987-89. Cardiovasc Ultrasound.

[B42] Eigenbrodt ML, Bursac Z, Eigenbrodt EP, Couper DJ, Tracy RE, Mehta JL (2004). Mathematical estimation of the potential effect of vascular remodelling/dilatation on B-mode ultrasound intima-medial thickness. Q J M.

[B43] Pasterkamp G, Galis ZS, Kleijn DPV (2004). Expansive arterial remodeling: location, location, location. Arterioscl Thromb Vasc Biol.

[B44] Masuda H, Zhuang YJ, Singh TM, Kawamura K, Murakami M, Zarins CK, Glagov S (1999). Adaptive remodeling of internal elastic lamina and endothelial lining during flow-induced arterial enlargement. Arterioscler Thromb Vasc Biol.

[B45] Lu X, Zhao JB, Wang GR, Gregersen H, Kassab GS (2001). Remodeling of the zero-stress state of femoral arteries in response to flow overload. Am J Physiol Heart Circ Physiol.

[B46] Tracy RE (2003). The role of aging in atherosclerosis: the sequestration hypothesis.

[B47] Al-Shali K, House AA, Hanley AJG, Kahn HMR, Harris SB, Mamakeesick M, Zinman B, Fenster A, Spence JD, Hegele RA (2005). Differences between carotid wall morphological phenotypes measured by ultrasound in one, two, and three dimensions. Atherosclerosis.

[B48] Iannuzzi A, De Michele M, Panico S, Celentano E, Tang R, Bond MG, Saccheti L, Zarrilli F, Galasso R, Mercuri M, Rubba P (2003). Radical-trapping activity, blood pressure, and carotid enlargement in women. Hypertension.

[B49] Sharrett AR, Coady SA, Folsom AR, Couper DJ, Heiss G (2004). Smoking and diabetes differ in their associations with subclinical atherosclerosis and coronary heart disease--the ARIC Study. Atherosclerosis.

[B50] Lorenz MW, Markus HS, Bots ML, Rosvall M, Sitzer M (2007). Prediction of clinical cardiovascular events with carotid intima-media thickness: a systemic review and meta-analysis. Circulation.

[B51] Glagov S, Weisenberg E, Zarins CK, Stankunavicius R, Kolettis GJ (1987). Compensatory enlargement of human atherosclerotic coronary arteries. N Engl J Med.

[B52] Hunt KJ, Evans GW, Folsom AR, Sharrett AR, Chambless LE, Tegeler CH, Heiss G (2001). Acoustic shadowing on B-mode ultrasound of the carotid artery predicts ischemic stroke: the Atherosclerosis Risk in Communities (ARIC) study. Stroke.

[B53] Paynter N, Coresh J (2006). Effect of outcome misclassification on risk prediction: can the area under the receiver-operator curve (AUC) always reach 1?. Am J Epidemiol.

